# Identification of Fatty Acid Metabolism-Related lncRNAs as Biomarkers for Clinical Prognosis and Immunotherapy Response in Patients With Lung Adenocarcinoma

**DOI:** 10.3389/fgene.2022.855940

**Published:** 2022-04-08

**Authors:** Helin Wang, Junwei Cui, Jian Yu, Jian Huang, Mingying Li

**Affiliations:** ^1^ Departments of Oncology, The First Affiliated Hospital of Xinxiang Medical University, Henan, China; ^2^ Departments of Tuberculosis, The First Affiliated Hospital of Xinxiang Medical University, Henan, China; ^3^ Departments of Pathology, The First Affiliated Hospital of Xinxiang Medical University, Henan, China

**Keywords:** fatty acid metabolism, lncRNA, nomogram, prognosis, lung adenocarcinoma

## Abstract

Lung adenocarcinoma (LUAD) is one of the most common malignant tumors with poor prognosis. Fatty acid metabolism is associated with cancer progression and a poor prognosis. We searched for long noncoding RNAs (lncRNAs) associated with fatty acid metabolism to predict the overall survival (OS) of patients with LUAD. We obtained lncRNA expression profiles and clinical follow-up data related to fatty acid metabolism in patients with LUAD from The Cancer Genome Atlas and Molecular Signatures database. Patients were randomly divided into training, experimental, and combination groups. Least absolute shrinkage and selection operator (LASSO) regression and Cox regression models were used to construct fatty acid metabolism-related prognostic markers, Kaplan-Meier analysis was used to compare the prognosis of each group, and receiver operating characteristic (ROC) analysis was used to evaluate the accuracy of the prognostic model. We used the pRRophetic algorithm to assess the treatment response based on the half-maximal inhibitory concentration (IC50) of each sample in the Genomics of Cancer Drug Sensitivity (GDSC) database. A fatty acid metabolism-related prognostic marker containing seven lncRNAs was constructed to predict OS in LUAD. In the training, test and combination groups, the patients were divided into high- and low-risk groups according to a formula. K–M analysis showed that patients in the high-risk group had poorer prognosis, with significant differences in the subgroup analysis. ROC analysis showed that the predictive ability of the model was more accurate. A clinical prediction nomogram combining lncRNA and clinical features was constructed to accurately predict OS and had high clinical application value. Therapeutics were screened based on the IC50 values of each sample in the GDSC database. We found that A.443654, AUY922, AZ628, A.770041, AZD.0530, AMG.706, and AG.014699 were more effective in high-risk patients. We constructed a 7-lncRNA prognostic model to predict the OS of patients with LUAD. In addition, the predictive nomogram model based on our established seven fatty acid metabolism-related lncRNA signatures provides better clinical value than that of the traditional TNM staging system in predicting the prognosis of patients with LUAD and presents new insights for personalized treatment.

## 1 Introduction

Lung cancer is the leading cause of cancer-related deaths worldwide (Siegel et al., 2020). Adenocarcinoma is one of the main pathological types of lung cancer (Li et al., 2020). Since targeted therapy and immunotherapy have been incorporated in the treatment of advanced and locally advanced lung adenocarcinoma (LUAD), extensive progress has been made in tumor treatment. However, only a minority of patients with LUAD achieve long-term survival (Zhang et al., 2019). Therefore, it is necessary to identify markers to stratify patients with lung cancer to obtain better treatment effects and utilize medical resources more efficiently.

Long non-coding RNAs (lncRNAs) are non-protein-coding RNAs that are at least 200 nucleotides in length (Ponting et al., 2009). With the development of high-throughput sequencing technology, an increasing number of lncRNAs have been reported as prognostic biomarkers for many cancers, including pancreatic, hepatocellular, and lung cancers (Peng and Jiang 2016; Zhao et al., 2017; Xie et al., 2018). Fatty acids in the human body play important roles in cellular metabolism, proliferation, and signal transduction (Reczynska et al., 2020). The fatty acid signaling pathway is one of the most important pathways involved in tumorigenesis and development. Cancer cells consume synthetic lipids as an energy source for proliferation, growth, survival, invasion, and angiogenesis (Amiri et al., 2018). Several studies have shown that fatty acid metabolism (FAM)-related lncRNAs are associated with the proliferation and differentiation of lung adenocarcinoma cells. High expression of FAM83A-AS1 in LUAD is closely associated with poor overall survival (OS) and progression-free survival (PFS) (Wang et al., 2021). FAM83A-AS1 promotes through the HIF-1α/glycolysis axis Proliferation and differentiation of LUAD (Chen et al., 2022). lncRNA LINC01116 is overexpressed in LUAD and promotes tumor proliferation and metastasis (Zeng et al., 2021). LINC00578 expression was closely associated with the survival of patients with LUAD (Zhao et al., 2018). Lipid metabolism-related mRNA in LUAD patients gene signatures are significantly associated with their diagnosis and prognosis (Li et al., 2020). However, prognostic markers based on FAM-related lncRNAs have not been investigated in LUAD.

This study aims to construct lncRNA signatures and nomograms related to FAM using a bioinformatics approach to improve the ability to predict OS in patients with LUAD. In addition, the FAM-related lncRNAs model was used as the target to explore potential therapeutic drugs to find new modes of immunotherapy.

## 2 Materials and Methods

### 2.1 Data Collection and Processing

Transcriptomic data, relevant clinical information, and mutation data of patients with LUAD were obtained from the TCGA database (https://portal.gdc.cancer.gov/). The downloaded files were processed using the R package. Unqualified data were converted and eliminated. All the data were calibrated, normalized, and log2-transformed. There were a total of 500 patients with 594 samples (59 normal and 535 tumor samples). To reduce statistical bias, patients with missing OS values were excluded. All the data were randomly divided into training and validation groups. This study was approved by the Ethics Committee of the First Affiliated Hospital of Xinxiang Medical University.

### 2.2 Differentially Expressed Gene Screening

Differential expression analysis of lncRNAs and mRNAs was performed between tumor tissue and normal tissue using the “ edgeR” R package. Differentially expressed genes were identified following the criteria of adjusted *p* < 0.05 and |Log_2_FC| > 1.0. Lists of significantly up- or downregulated genes were saved as XLS files. Furthermore, we used the “edgeR” package, a Bioconductor package, to identify differentially expressed lncRNAs (difflncRNAs) and mRNAs (diffmRNAs) based on TCGA dataset.

### 2.3 Screening of Fatty Acid Metabolism mRNA and its Related lncRNA mRNA

In total, 532 FAM-related mRNAs (FAM mRNAs) were obtained from the GSEA database (https://www.gsea-msigdb.org/gsea/msigdb/index.jsp). The intersection of FAM mRNA and diffmRNA was obtained to determine the expression matrix of FAM diffmRNA in LUAD. The “limma” R package was used to analyze lncRNAs whose difflncRNAs were significantly associated with a gene in FAM diffmRNA (|corFilter| > 0.4, *p* < 0.001).

### 2.4 Construction and Validation of a Prognostic Signature Model

An FAM-related lncRNA model was constructed using the training set, and the established model was validated using the entire dataset and test set. As shown in Table 1, there were no significant differences in clinical characteristics between the two datasets (*p* > 0.05). Combined with the LUAD survival information in TCGA, we performed a univariate Cox regression analysis on the screened FAM-related difflncRNAs. Using the R package “glmnet” for least absolute shrinkage and selection operator (LASSO) Cox regression, we found that 12 FAM-related lncRNAs were significantly associated with tumor OS. To assess factors independently associated with prognosis, we performed a multivariate Cox regression analysis. Ultimately, seven FAM-related lncRNAs were identified as prognostic factors. Patients were divided into low- and high-risk groups based on the median risk score. Risk score was calculated as follows:

Risk score = exp⁡r(ln⁡cRNA1)× coef(ln⁡cRNA1)

+ exp⁡r(ln⁡cRNA2) × coef(ln⁡cRNA2)+ ..+ exp⁡r(ln⁡cRNAn) × coef(ln⁡cRNAn)

where coef is the coefficient and expr is the expression level of lncRNA.

**TABLE 1:** Patients’ clinical characteristics.

Characteristics		All Group		Test Group		Training Group		Pvalue
		Number	Percentage (%)	Number	Percentage (%)	Number	Percentage (%)	
**Age**	≤65	237	47.40	114	45.97	123	48.81	0.7088
	>65	253	50.60	127	51.21	126	50	
	unknow	10	2	7	2.82	3	1.19	
**Gender**	FEMALE	270	54	133	53.63	137	54.37	0.9399
	MALE	230	46	115	46.37	115	45.63	
**Stage**	Stage I	268	53.60	134	54.03	134	53.17	0.5944
	Stage II	119	23.80	60	24.19	59	23.41	
	Stage III	80	16	40	16.13	40	15.87	
	Stage IV	25	5	9	3.63	16	6.35	
	unknow	8	1.60	5	2.02	3	1.19	
**T**	T1	167	33.40	81	32.66	86	34.13	0.7543
	T2	267	53.40	133	53.63	134	53.17	
	T3	45	9	25	10.08	20	7.94	
	T4	18	3.60	7	2.82	11	4.37	
	TX	3	0.60	2	0.81	1	0.40	
**M**	M0	332	66.40	172	69.35	160	63.49	0.1539
	M1	24	4.80	8	3.23	16	6.35	
	MX	140	28	65	26.21	75	29.76	
	unknow	4	0.80	3	1.21	1	0.40	
**N**	N0	324	64.80	162	65.32	162	64.29	0.9819
	N1	94	18.80	46	18.55	48	19.05	
	N2	69	13.80	34	13.71	35	13.89	
	N3	2	0.40	1	0.40	1	0.40	
	NX	10	2	4	1.61	6	2.38	
	unknow	1	0.20	1	0.40	0	0	

### 2.5 Functional Analysis

Analysis was performed using the R package “clusterProfiler” to annotate the functions of the differentially expressed genes. A *p*-value < 0.05 indicated a significant enrichment of functional annotations.

### 2.6 Exploration of Immunotherapy Modes

The R package “maftools” was used to analyze the mutation data. Tumor mutational burden (TMB) was calculated based on tumor-specific mutated genes. We used the tumor immune dysfunction and exclusion (TIDE) algorithm to predict the immunotherapy response.

### 2.7 Principal Component Analysis and Kaplan-Meier Survival Analysis

PCA of the high- and low-risk groups was performed using four datasets (Fig. difference between. We performed K–M survival analysis using the R packages “survMiner” and “Survive” to assess the difference in OS between the high- and low-risk groups.

### 2.8 Exploring Potential Therapeutic Drugs Targeting Fatty Acid Metabolism-Related lncRNAs Models

To explore potential clinical drugs for the treatment of LUAD, we used the R package “pRRophetic” to predict the IC50 values of the compounds obtained from the Genomics of Cancer Drug Sensitivity (GDSC) website according to the LUAD dataset in TCGA database.

### 2.9 Independence of the Fatty Acid Metabolism-Related lncRNAs Model

To test whether prognostic patterns were independent variables for other clinical characteristics (sex, age, and TNM stage), univariate and multivariate Cox regression analyses were performed in patients with LUAD.

### 2.10 Establishing and Proving a Predictive Nomogram

A nomogram was constructed and its predictive power for 1-, 3-, and 5-years OS was predicted. A calibration plot was used to illustrate the agreement between the actual results and model predictions, which was based on the Hosmer-Lemeshow test.

## 3 Results

### 3.1 Identification of Fatty Acid Metabolism-Related lncRNAs

**FIGURE 1:**
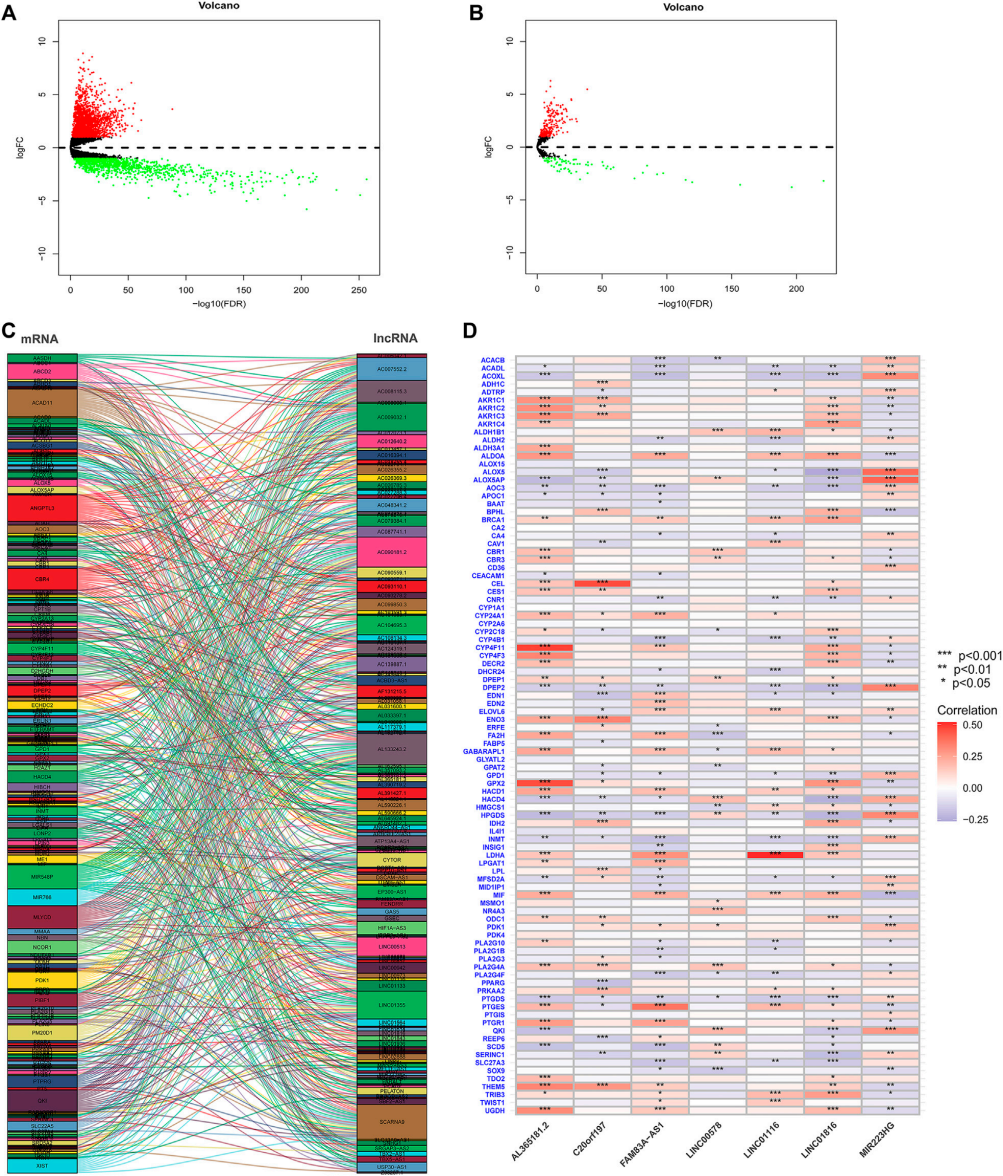
**(A)** A total of 19,573 differentially expressed mRNAs were analyzed to obtain 3569 significantly differentially expressed mRNAs (diffmRNAs) (|log_2_FC|>1, *p* < 0.05). **(B)** Differential expression analysis of 14,056 lncRNAs revealed 295 lncRNAs (difflncRNAs) that were significantly differentially expressed (|log_2_FC|>1, *p* < 0.05). **(C)** Sankey plot of lncRNAs with a significant correlation between difflncRNA and a gene in FAMdiffmRNA (|corFilter|>0.4, *p* < 0.001). **(D)** Heatmap of the correlation between difflncRNA and FAMdiffmRNA expression.

A total of 56,461 RNA matrix expressions were extracted from the TCGA database, including 19,573 mRNAs and 14,056 lncRNAs. Using the “edgeR” R package to analyze differentially expressed genes, a total of 3,569 significantly diffmRNAs (Figure 1A, 295difflncRNAs (Figure 1B) were obtained (|log_2_FC|>1, *p* < 0.05). A total of 532 FAM-related mRNAs were obtained from the GSEA database. DiffmRNA and FAMmRNA were intersected to obtain 95 FAM-related diffmRNA (FAMdiffmRNA) expression matrices. The “limma” R package was used to analyze the lncRNAs with a significant correlation between difflncRNA and a gene in FAMdiffmRNA (|corFilter|>0.4, *p* < 0.001) (Figures 1C, 131FAM-related difflncRNAs were obtained. Figure 1D shows the correlation between difflncRNA and FAMdiffmRNA expression levels.

### 3.2 Construction and Validation of a Risk Model for Fatty Acid Metabolism-Related lncRNAs in Lung Adenocarcinoma Patients

**FIGURE 2:**
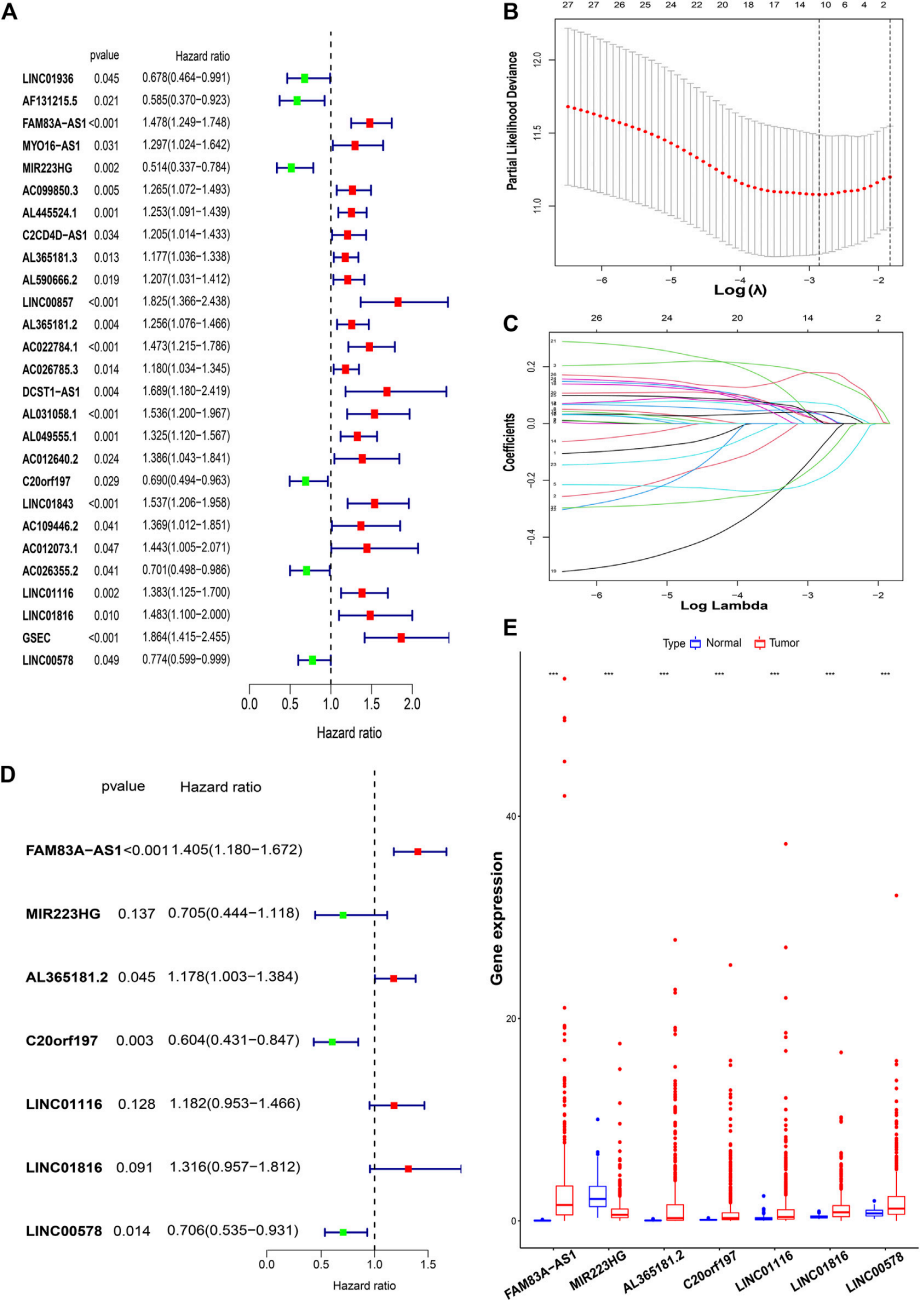
Construction of a risk model for FAM-related lncRNAs in LUAD patients. **(A)** Univariate Cox regression analysis of FAM-related lncRNAs significantly associated with clinical prognosis. **(B** and **C)** LASSO-Cox regression algorithm was applied to select genes based on minimum criteria to construct risk signatures. **(D)** Multivariate Cox regression analysis of independent prognostic factors. **(E)** These seven lncRNAs were significantly differentially expressed between normal tissues and tumor tissues. ***,*p* < 0.001.

Univariate Cox regression analysis identified FAM-related prognostic lncRNAs from 131 FAM-related difflncRNAs and obtained 27 FAM-related difflncRNAs that were significantly associated with OS, including six protective factors and 21 risk factors (Figure 2A). To identify the best prognostic genes, we applied the LASSO-Cox regression algorithm to the 27 prognostic-related genes (Figures 2B,C) and selected 12 genes according to the minimum criteria to construct the risk signature. Multivariate Cox hazard regression was then used to assess the independent prognostic values of the 12 candidate prognostic genes. Multivariate COX regression analysis demonstrated that 7 lncRNAs were associated with prognosis (Figure 2D). The risk score was calculated as follows [FAM83A-AS1 × (0.339909)] + [MIR223HG × (−0.349849)] + [AL365181.2 × (0.164038)] + [C20orf197 × (-0.503650)] + [LINC01116 × 0.167043)] + [LINC01816 × (0.274919)] + [LINC00578 × (−0.348438)]. Patients were divided into high- and low-risk groups according to the median risk score. According to the Cox model, seven genes were selected as prognostic factors. Four genes (FAM83A-AS1, AL365181.2, C20orf197, and LINC00578) were independent significant prognostic factors (Figure 2D). These seven lncRNAs were significantly differentially expressed between normal tissues and tumor tissues (Figure 2E).

**FIGURE 3:**
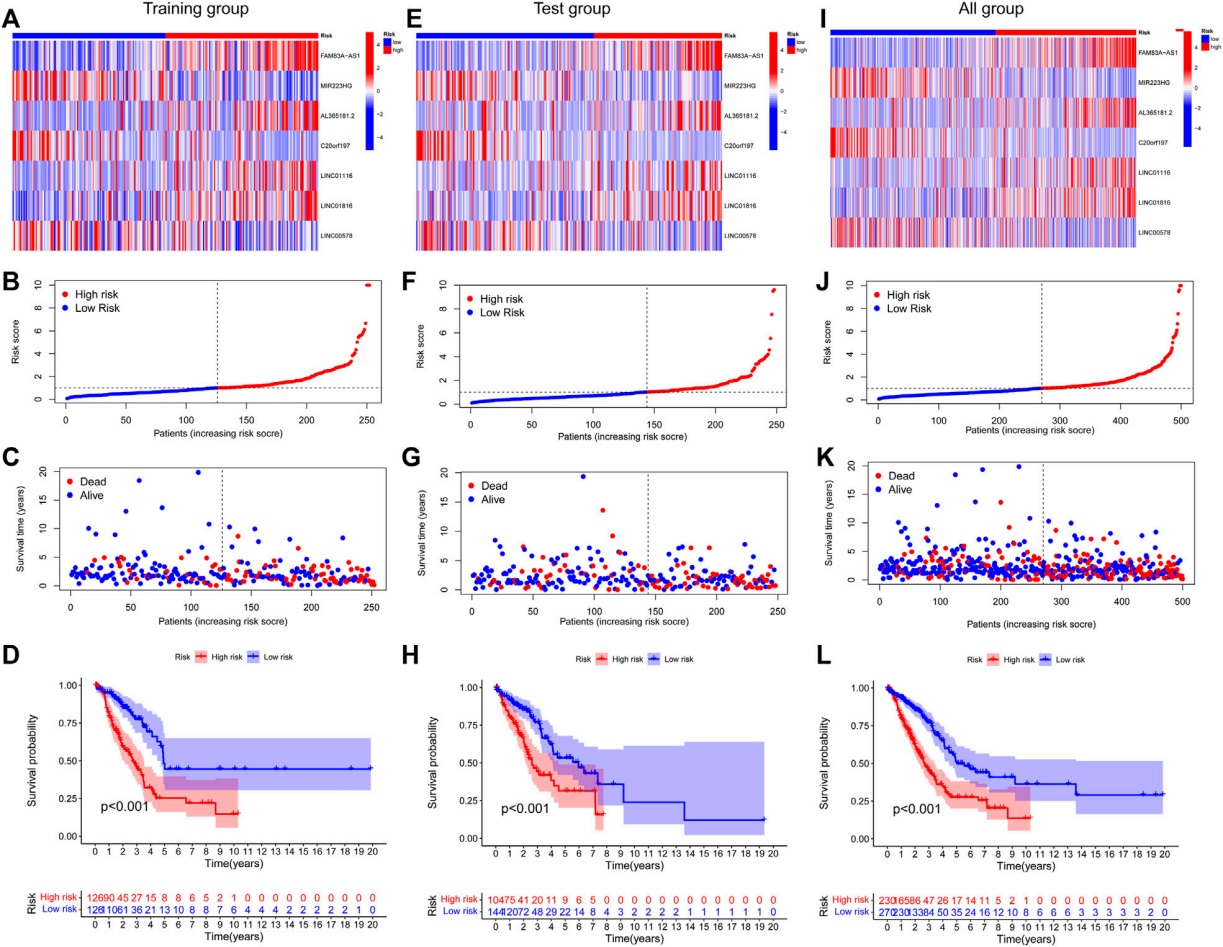
Prognostic value of risk patterns of seven FAM-associated lncRNAs. **(A,E and I)** Training group, test group, and whole group cluster analysis heatmap showing the expression levels of seven prognostic factors per patient. **(B)** F and J, distribution of risk scores for the training group, test group, and whole group based on the FAM-associated lncRNAs model. **(C,G and K)** Differences in survival status and survival time patterns between the training, test, and whole groups of high- and low-risk groups.**(D,H and L)** K–M survival curves for OS in the training group, test group, and the entire cohort of patients in the high- and low-risk groups.

Finally, 7-lncRNAs were selected to construct the model. Figure 3 shows the ability of the seven lncRNAs to predict OS. A heatmap of prognostic marker scores in the training group (Figure 3A), the distribution of patients in different risk groups (Figure 3B), and patients’ OS status (Figure 3C) are shown. K–M analysis of the training group showed that the OS of the low-risk group was significantly better than that of the high-risk group (*p* < 0.05, Figure 3D). In addition, we performed a validation analysis of the 7-lncRNA signature in the experimental and combined groups, yielding similar results (Figure 3E–L).

### 3.3 Further Validation of the Grouping Ability of the Fatty Acid Metabolism-Related lncRNAs Model by Principal Component Analysis

**FIGURE 4:**
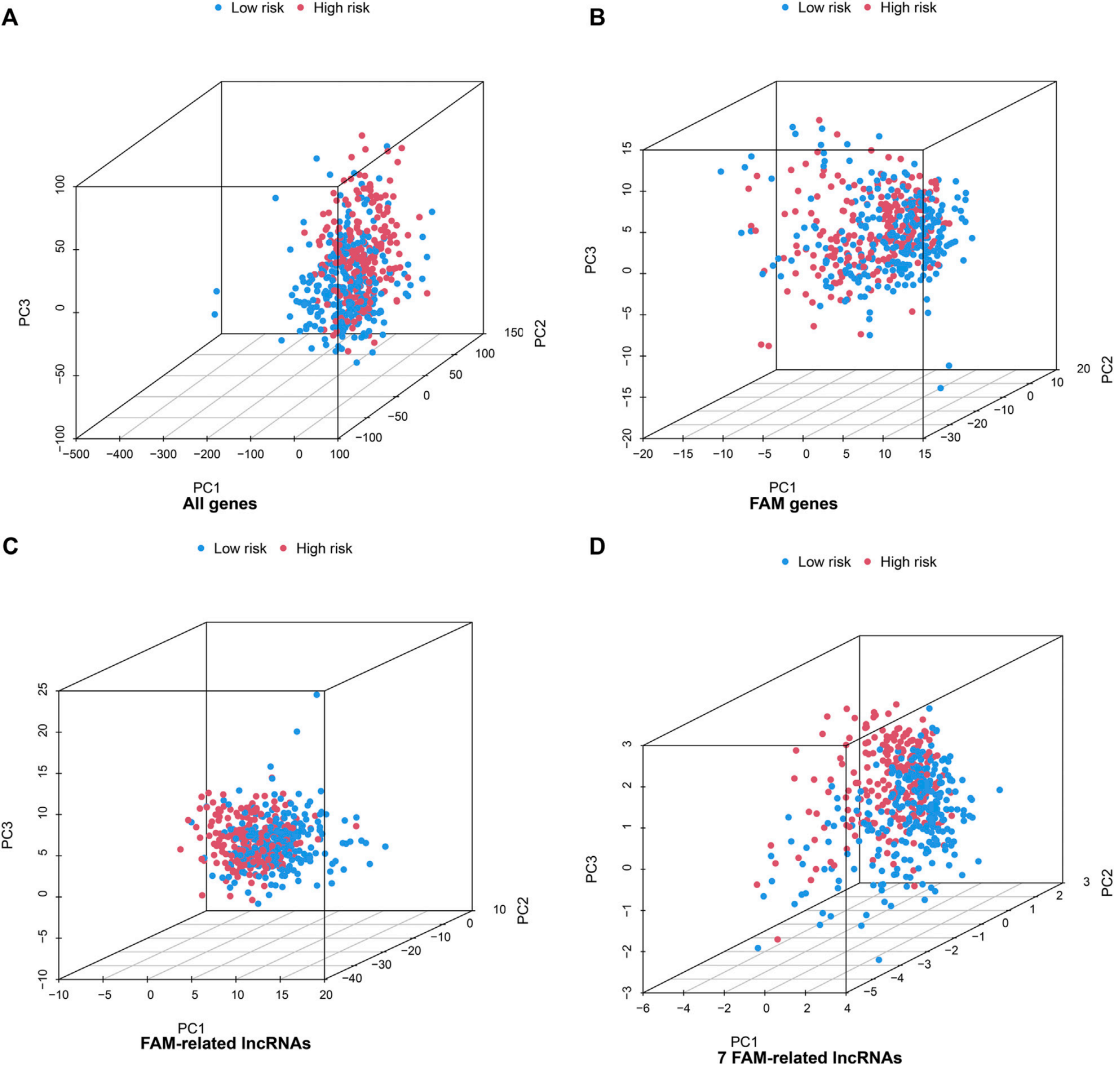
Principal component analysis between the high- and low-risk groups based on **(A)** all gene expression profiles, **(B)** FAM genes **(C)** FAM-related lncRNAs, and **(D)** risk model based on the 7 FAM-related lncRNAs in the entire TCGA set.

PCA of the high- and low-risk groups was performed using four datasets (Figure 4). Figures 4A–C show that the distributions of the high- and low-risk groups were relatively widespread. The analysis results according to the risk model we constructed showed that the low- and high-risk groups had different distributions (Figure 4D). This shows that the risk model can distinguish between low- and high-risk groups.

### 3.4 Assessment of Tumor Immune Microenvironment and Tumor Immunotherapy Response Using the Fatty Acid Metabolism-Associated lncRNA Model

**FIGURE 5:**
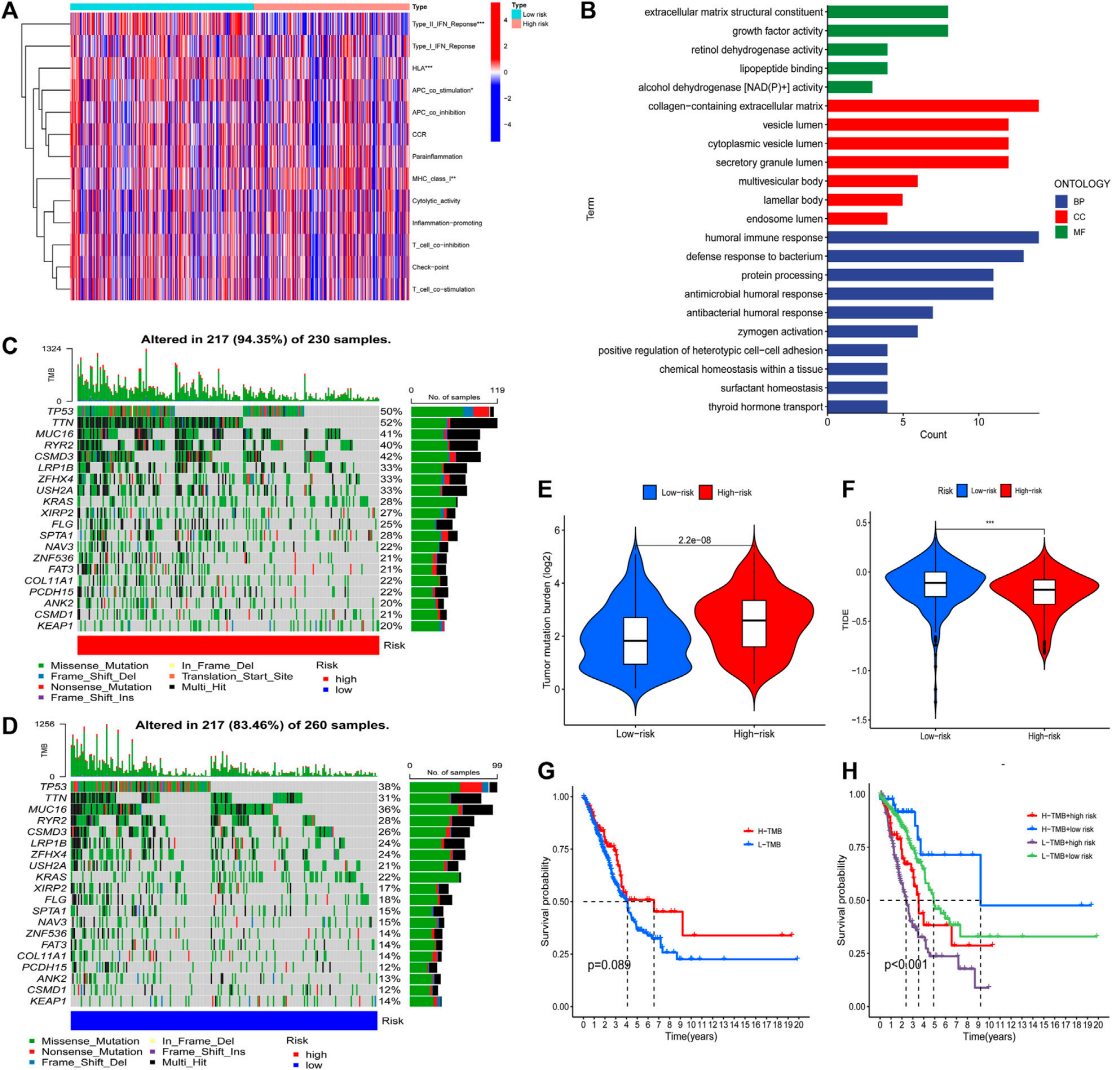
The tumor immune microenvironment and cancer immunotherapy response were assessed using a model of FAM-associated lncRNAs across the TCGA genome. **(A)** Indicator criteria for each patient’s immune index. **(B)** GO enrichment analysis. **(C** and **D)** Waterfall plots showing mutation information for genes with high mutation frequency in high- **(C)** and low-risk groups **(D)**. **(E)** Differences in tumor mutational burden between high- and low-risk patients. **(F)** Differences in TIDE between high- and low-risk patients. **(G)** K–M curve analysis of patient OS according to TMB. **(H)** K–M curve analysis of patient OS according to model risk of TMB and FAM-related lncRNAs. ***,*p* < 0.001.

Based on the FAM-related lncRNA model, the enrichment levels and activities of various immune cells, immune pathways, and immune functions in 500 patients with LUAD were further analyzed. There were significant differences in the expression of immune indices Type_II_IFN_Reponse, HLA, APC_co_stimulation, and MHC_class_I between the low- and high-risk groups (Figure 5A). We performed Gene Ontology enrichment analysis to further explore the underlying molecular mechanisms of risk models based on FAM-related lncRNAs, revealing many biological processes related to humoral immunity and protein production (Figure 5B). We then analyzed the mutation data. Mutations were stratified according to the constructed risk model. Figures 5C,D show the top 20 driver genes with the highest mutation frequencies between the high- and low-risk groups. It can be seen that the mutation frequency in the high-risk group is significantly higher than in the low-risk group. TMB scores were calculated from the TGCA somatic mutation data. The high-risk group had significantly higher TMB than that of the low-risk group (*p* < 0.001), indicating that the FAM-based classification index was highly correlated with TMB (Figure 5E). Next, we investigated the correlation between FAM-associated lncRNA models and immunotherapy biomarkers. Unexpectedly, we found that the high-risk group was more likely to respond to immunotherapy than the low-risk group, suggesting that this FAM-based classification index could serve as a predictor of TIDE (Figure 5F).

Finally, K–M curve analysis was performed for patient OS according to TMB. As shown in Figure 5G, there was no significant difference between the patients in the high and low TMB groups. Therefore, we examined whether the FAM-associated lncRNA model could predict OS outcomes better than TMB alone. Patients with high and low TMB in the low-risk group (defined as TMB high/low and TMB low/low, respectively) exhibited a higher risk for improved OS (Figure 5H). Interestingly, patients in the high- and low-risk groups with low TMB (TMB low/high and TMB low/low, respectively) had worse survival outcomes than those in the high- and low-risk groups with high TMB (TMB high/high and TMB high/low, respectively). The low-risk group with high TMB had the best prognosis among those of the other three groups. In conclusion, the risk model of FAM-associated lncRNAs has greater prognostic significance than that of TMB.

### 3.5 Construction and Evaluation of a Prognostic Risk Model and Predictive Nomogram of Fatty Acid Metabolism-Related lncRNAs in Patients With Lung Adenocarcinoma

**FIGURE 6:**
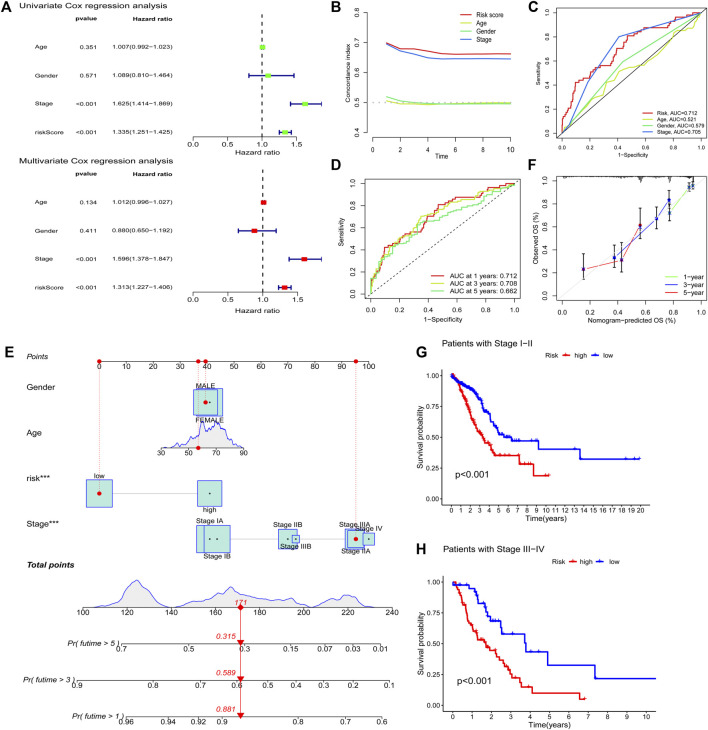
Evaluating prognostic risk models for FAM-associated lncRNAs and clinical characteristics in LUAD patients. **(A)** Univariate and multivariate analysis of clinical characteristics and risk scores. **(B)** Concordance index of risk score with clinical characteristics. **(C)** ROC curves of clinical features and risk scores. **(D)** ROC curves for 1-, 2-, and 3-years OS. **(E)** Nomogram predicting the probability of OS at 1, 2, and 3 years. **(F)** Calibration plot predicting the agreement between observed and predicted rates of OS at 1, 2, and 3 years. **(G** and **H)** K–M curve analysis of patient OS according to FAM-related lncRNAs model risk in stage I-II and stage III-IV patients, respectively. ***,*p* < 0.001.

To assess whether the risk model of the seven FAM-related lncRNAs had independent prognostic features for LUAD, we performed univariate and multivariate Cox regression analyses. Univariate Cox regression analysis showed that the hazard ratio and 95% confidence interval of clinical stage were 1.625 and 1.414–1.869 (*p* < 0.001), respectively. The hazard ratios and 95% confidence intervals for the hazard scores were 1.335 and 1.251–1.425, respectively (*p* < 0.001). After multivariate Cox regression analysis, the hazard ratio and 95% confidence interval of the clinical stage were 1.596 and 1.378–1.847 (*p* < 0.001). Hazard ratios and 95% confidence intervals for hazard scores were 1.313 and 1.227–1.406 (*p* < 0.001), respectively (Figure 6A). This suggests that the risk model and clinical stage of the seven FAM-related lncRNAs are independent prognostic factors. To better assess the specificity and sensitivity of the risk score in predicting prognosis in patients with LUAD, we assessed the compliance index of the risk score and the area under the ROC curve (AUC). Over time, the compliance index of the risk score was greater than those of age, sex, and stage, suggesting that the risk score could better predict the prognosis of LUAD (Figure 6B). The AUC of the risk score was also higher than those of age, sex, and stage, indicating that the seven FAM-related lncRNAs were more reliable in the prognostic risk model of LUAD (Figure 6C). The AUCs at 1, 2, and 3 years were 0.712, 0.708, and 0.662, respectively (Figure 6D), suggesting that the risk model we constructed could better predict the prognosis.

We predicted the 1-, 2-, and 3-years OS rates by constructing nomograms incorporating risk classes and clinical characteristics (Figure 6E). Furthermore, there was a good agreement between the observed and predicted OS rates at 1, 2, and 3 years (Figure 6F). Finally, we examined the ability of the FAM-associated lncRNA model to predict the OS at different stages. The results showed that the OS of the low-risk group was significantly better than that of the high-risk group (*p* < 0.001) in both stage I–II and stage III–IV groups (Figures 6G,H).

### 3.6 Screening Potential Drugs by Targeting Fatty Acid Metabolism-Related lncRNA Models

**FIGURE 7:**
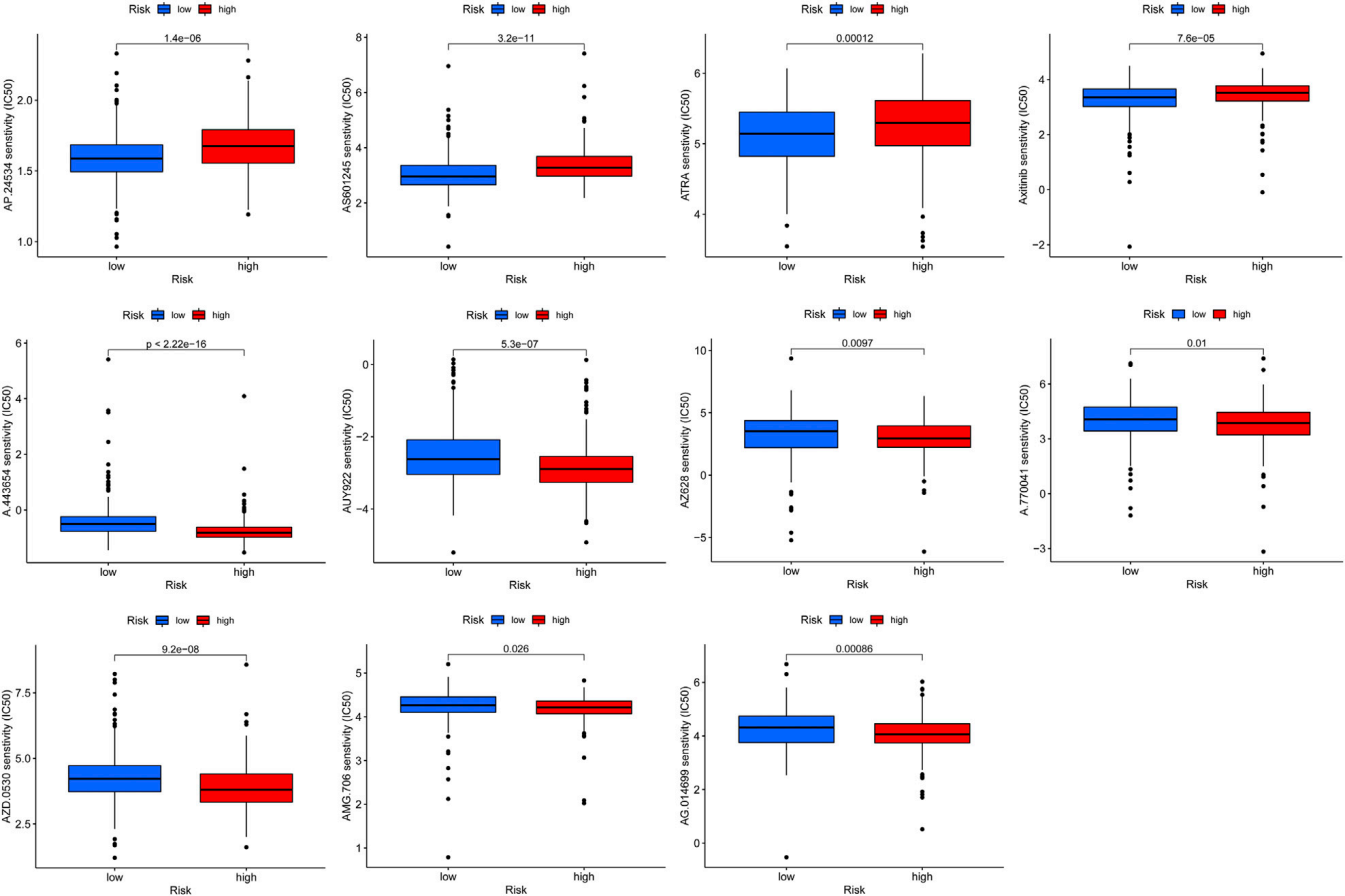
Screening potential drugs by targeting FAM-related lncRNAs models.

According to the above analysis, the prognosis of patients in the high-risk group was poor. To screen for potential drugs targeting the FAM-associated lncRNA model to treat patients with LUAD, we used the pRRophetic algorithm to evaluate treatments based on the half-maximal inhibitory concentration (IC50) of each sample in the GDSC database. We found that the estimated IC50s of the 11 compounds differed significantly between the two groups, suggesting greater sensitivity to these drugs when the high-risk group had lower IC50s than that of the low-risk group (Figure 7). Therefore, A.443654, AUY922, AZ628, A.770041, AZD.0530, AMG.706, and AG.014699 were more effective for high-risk patients.

## 4 Discussion

The TNM staging system is currently the gold standard for evaluating tumor prognosis; however, it has certain limitations. Patients with the same stage often have different treatment effects and prognoses (Choi et al., 2017). Therefore, identifying new prognostic markers is a challenge in medical research. Owing to the development of bioinformatics technology, lncRNA-based signatures have attracted considerable attention due to their higher prediction accuracy (Liu et al., 2020).

After univariate and multivariate Cox regression analyses, we identified four genes (FAM83A-AS1, AL365181.2, C20orf197, and LINC00578) as significant independent prognostic factors. Previous studies have shown that these four lncRNAs are associated with the prognosis and progression of LUAD. The lncRNA FAM83A-AS1 promotes lung adenocarcinoma cell migration, invasion, and tumor progression (Shi et al., 2019; Xiao et al., 2019; Wang et al., 2021). AL365181.2 is an immune-related lncRNA prognostic marker in LUAD (Wu et al., 2021), while LINC00578 is a prognostic marker (Wang et al., 2019). C20orf197 is a prognostic marker of ferroptosis and iron metabolism-related lncRNAs in LUAD (Yao et al., 2021).

TMB is a somatic mutational burden associated with the emergence of neoantigens that trigger antitumor immunity (Allgauer et al., 2018). Recent studies have shown that TMB is an effective biomarker for predicting response to PD-L1 therapy (Topalian et al., 2016). We found that the high-risk group had more TMB than the low-risk group. In addition, an increasing number of studies have used the TIDE prediction score, a computational framework for immunotherapy prediction and its predictive function has been successfully validated (Jiang et al., 2018). We found that the high-risk group was more likely to respond to immunotherapy than the low-risk group. Based on the above results, we infer that this predictive model can provide reliable immune markers for tumor therapy. Furthermore, our study provides new insights into the molecular biological mechanisms of LUAD FAM-associated lncRNAs.

We targeted lncRNAs related to FAM and screened for therapeutic drugs based on the IC50 of each sample in the GDSC database. We found that A.443654, AUY922, AZ628, A.770041, AZD.0530, AMG.706, and AG.014699 were more effective in high-risk group patients, which provides a new potential antitumor therapy.

An increasing number of nomograms have been used to assess tumor prognosis (Balachandran et al., 2015). The advantage of the nomogram is individualized risk assessment based on patient clinical or disease characteristics (Chen et al., 2020). In this study, we constructed a prognostic nomogram incorporating clinical features and FAM-related lncRNA signals. The results showed that patients with high-risk scores had shorter OS than those with low-risk scores. The nomograms we established show that the observed and predicted rates at 1-, 3-, and 5-years are perfect consistency. Finally, the observations are in excellent consistency with the 1-, 3-, and 5-years predictions. The risk model based on 7 FAM-related lncRNAs is quite accurate, and this predictive model can identify new biomarkers for follow-up studies. Furthermore, ROC curve analysis confirmed that 7-lncRNA signaling was a highly sensitive and specific prognostic marker for non-small cell lung cancer. More importantly, the accuracy and clinical value of our nomogram for predicting prognosis were higher than those of the traditional TNM staging systems.

Although our nomogram has many advantages, it has certain limitations. First, our study population mainly consisted of patients from international databases and was internally validated. We also need to confirm the accuracy of lncRNA signatures associated with FAM in other databases and local data for external validation. Second, some FAM-related lncRNAs are rarely reported in the literature on PPI, and the mechanism of FAM-related lncRNAs in non-small cell lung cancer still needs to be elucidated *in vivo* and *in vitro*.

In conclusion, we constructed a 7-lncRNA prognostic model to predict the OS of patients with non-small cell lung cancer. In addition, the predictive nomogram model based on our established seven fatty acid metabolism-related lncRNA signatures provides better clinical value than that of the traditional TNM staging system in predicting the prognosis of patients with non-small cell lung cancer and presents new insights for personalized treatment.

## Data Availability

Publicly available datasets were analyzed in this study. This data can be found here: https://portal.gdc.cancer.gov/.
